# Metagenomics and digital cell modeling facilitate targeted high‐throughput sorting of anaerobic hydrogen‐producing microorganisms

**DOI:** 10.1002/imt2.70082

**Published:** 2025-09-21

**Authors:** Jianfeng Liu, Wei Xing, Xingyang Zhang, Nengyao Xu, Ran Xu, Junsha Gong, Jia Zhang, Fengai Yang, Shuang Gao, Yanan Hou, Yongping Shan, Bin Liu, Qianqian Yuan, Aijie Wang, Nanqi Ren, Cong Huang

**Affiliations:** ^1^ Key Laboratory of Environmental Biotechnology, Research Center for Eco‐Environmental Sciences Chinese Academy of Sciences Beijing China; ^2^ National Technology Innovation Center of Synthetic Biology, Tianjin Institute of Industrial Biotechnology Chinese Academy of Sciences Tianjin China; ^3^ School of Environmental and Municipal Engineering Tianjin Chengjian University Tianjin China; ^4^ College of Biotechnology Tianjin University of Science and Technology Tianjin China; ^5^ Key Laboratory of Environmental Nanotechnology and Health Effects, Research Center for Eco‐Environmental Sciences Chinese Academy of Sciences Beijing China; ^6^ State Key Laboratory of Regional Environment and Sustainability, Research Center for Eco‐Environmental Sciences Chinese Academy of Sciences Beijing China; ^7^ University of Chinese Academy of Sciences Beijing China; ^8^ School of Civil and Environmental Engineering Harbin Institute of Technology (Shenzhen) Shenzhen China

## Abstract

This study proposes a novel strategy that prioritizes functional recognition, followed by targeted high‐throughput sorting, to enable the comprehensive, rapid, and efficient acquisition of target microorganisms. Using metagenomic sequencing and binning analysis, we identified 215 potential anaerobic hydrogen‐producing strains from 12 large‐scale biogas samples. Digital cell models were subsequently constructed from metagenome‐assembled genomes, which guided the design of 14 selective culture media for enriching these hydrogen‐producing bacteria. Flow cytometry‐based high‐throughput sorting successfully isolated 81 potential anaerobic hydrogen‐producing strains, achieving a target acquisition rate above 37% and a survival rate exceeding 70%. This method holds broad potential for the discovery and sorting of functional microorganisms across diverse environments and may ultimately facilitate the development of synthetic microbiomes for industrial applications.

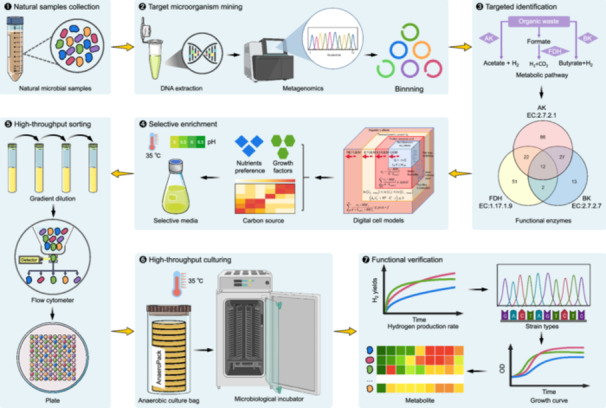

To the Editor,

Hydrogen is a clean‐burning fuel frequently heralded as the fuel of the future. The traditional hydrogen production system for natural mixed bacteria is often referred to as a “black box” [1]. Its complexity and instability result in inefficiencies such as insufficient substrate utilization, low hydrogen yield, and low hydrogen production rate, severely hindering industrial applications. With the rapid advancements in microbiome research, computational biology, and synthetic biology, scientists have started integrating different microbial strains to artificially construct efficient, stable, and controllable microbial communities, termed “synthetic microbiomes” [2]. The advent of synthetic microbiomes has transformed the approach from a “black box” to a “white box,” enabling the flexible regulation of microorganisms within each metabolic functional module. This innovation holds promise for establishing a novel modality for biohydrogen production. Notably, functional strains are the cornerstone of synthetic microbiomes. Traditional anaerobic bacteria sorting methods, such as tube rolling and plate streaking, are labor‐intensive and time‐consuming and do not support high‐throughput sorting. Furthermore, the current strategy for sorting hydrogen‐producing functional strains typically follows a “cultivation first, followed by sorting” approach [3]. The target strains are blindly enriched, cultured, and sorted, akin to “The Blind Men and the Elephant” fable, owing to the limited knowledge of the species, nutrient requirements, and growth factors of hydrogen‐producing bacteria in natural mixed bacterial samples [4]. Target strains cannot be obtained for culturable microorganisms without the appropriate culture medium and growth conditions [5]. Therefore, this strategy cannot comprehensively capture hydrogen‐producing bacteria.

To overcome the limitations of traditional sorting methods, we propose a novel strategy, “prioritizing functional recognition, followed by targeted high‐throughput sorting.” This approach establishes a high‐throughput workflow for the mining, identifying, cultivating, and sorting anaerobic hydrogen‐producing strains. High‐throughput 16S ribosomal ribonucleic acid (rRNA) gene sequencing and metagenomic techniques were employed to comprehensively mine and identify potential anaerobic hydrogen‐producing microorganisms from 12 large‐scale biogas samples (Figure 1). Subsequently, digital cell models were constructed based on the protein sequences of metagenome‐assembled genomes (MAGs) to guide the preparation of selective culture media tailored for hydrogen‐producing microorganisms. Finally, flow cytometry was performed for high‐throughput sorting, enabling rapid batch acquisition of target hydrogen‐producing functional strains. This approach lays the groundwork for the development of synthetic microbiomes for efficient biohydrogen production.

**Figure 1:**
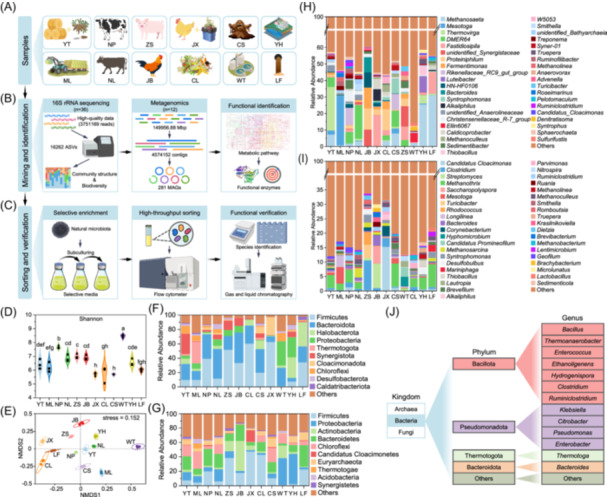
Experimental workflow and analysis of microbial communities. (A) Schematic of the 12 large‐scale biogas plant samples in China. The samples were collected from various biogas projects: YT from corn straw (Tonghua), ML from cassava residue and straw (Long'an), NP from cow manure (Pingdingshan), NL from cattle manure (Liangshan), JB from chicken manure (Chongqing), JX from chicken manure and kitchen waste (Xuzhou), CL from kitchen waste hydrolysate (Liu'an), CS from kitchen waste (Sanming), ZS from pig manure (Suining), WT from municipal wastewater (Tianjin), and YH from pharmaceutical wastewater (Huanggang). (B) The 16S ribosomal RNA (rRNA) gene high‐throughput sequencing and metagenomics analysis were employed to comprehensively mine and identify potential anaerobic hydrogen‐producing microorganisms. (C) Hydrogen‐producing microorganisms were selectively enriched, followed by high‐throughput sorting using flow cytometer. (D) Alpha diversity represented by Shannon index (*p* < 0.05). (E) Beta diversity of the microbial communities. Nonmetric multidimensional scaling analysis with Bray–Curtis distance matrix of the bacterial communities. (F) Community composition at the phylum level detected by 16S rRNA sequencing. (G) Community composition at the phylum level detected by metagenomics. (H) Community composition at the genus level detected via 16S rRNA sequencing. (I) Community composition at the genus level detected by metagenomics. (J) Commonly identified hydrogen‐producing microorganisms across samples.

### Mining of hydrogen‐producing microorganisms

In this study, 16S rRNA gene high‐throughput sequencing and metagenomic techniques were employed to mine potential anaerobic hydrogen‐producing microorganisms in samples from 12 large‐scale biogas plants. A total of 16,262 amplicon sequence variants were detected across all samples (Figure S1). The alpha diversities of the microbial community were evaluated using the Shannon indices (Figure 1D). Alpha diversity differed significantly among groups (Kruskal–Wallis *H* test, *p* < 0.05). Post hoc analysis revealed that WT showed the highest diversity, significantly exceeding all other groups (*p* < 0.05), while JX and CS had the lowest values and did not differ from each other (*p* > 0.05, Figure 1D). In terms of the beta diversity, nonmetric multidimensional scaling ordination based on the Bray–Curtis distance matrix revealed distinct visual clustering patterns among sample groups (stress = 0.152, Figure 1E).

We analyzed the relative abundance of the microbial communities at the phylum and genus levels. The top 10 dominant phyla identified through 16S rRNA gene high‐throughput sequencing and metagenomic analysis were highly similar (Figure 1F,G). However, some discrepancies were observed in the top 40 dominant genera (Figure 1H,I). Figure 1J provides a systematic summary of hydrogen‐producing microorganisms reported in previous studies [6, 7]. Notably, both analyses detected the genera *Bacteroides* and *Ruminiclostridium*. Additionally, metagenomics detected the genus *Clostridium*, highlighting its potential for mining functional microorganisms.

To further explore potential anaerobic hydrogen producers, gene sequences from the 12 large‐scale biogas plant samples were assembled using data binning technique. This process resulted in a total of 281 MAGs with completeness greater than 70% and contamination levels below 10%. These MAGs enabled the inference of functional categories, protein domains, metabolic potentials, and other genomic traits [8]. A phylogenetic tree of all MAGs was constructed to visualize their relationships (Figure S2). Detailed information regarding the completeness and contamination of each MAG is provided in Table S1. MAGs that are evolutionarily closely related often exhibit similar nutritional requirements and culture conditions, offering new possibilities for the targeted isolation of previously uncultured microorganisms.

### Identification of hydrogen‐producing microorganisms

By analyzing functional genes, that is, those associated with hydrogen production, we can rapidly identify potential hydrogen‐producing microorganisms. The core metabolic pathways involved in anaerobic fermentation for hydrogen production are illustrated in Figure 2A. These pathways include formate cleavage, acetate‐type fermentation, and butyrate‐type fermentation, and the corresponding functional enzymes are formate dehydrogenase (FDH, EC: 1.17.1.9), acetate kinase (AK, EC: 2.7.2.1), and butyrate kinase (BK, EC: 2.7.2.7), respectively. Additionally, numerous competitive metabolic pathways generate reduction byproducts, such as propionate, ethanol, and lactate. These byproducts consume significant amounts of NADH, a crucial molecule for hydrogen production, thereby reducing the overall hydrogen yield [9].

**Figure 2:**
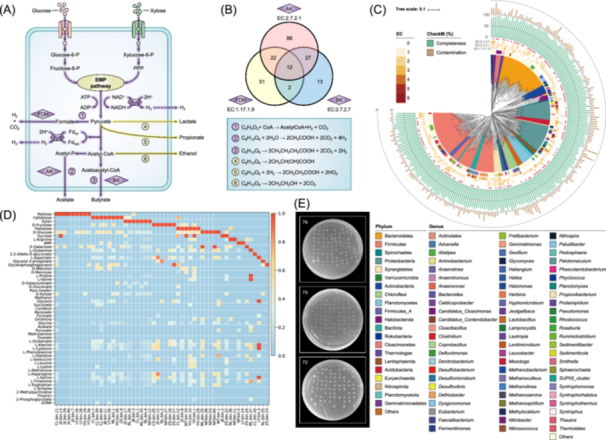
Identification, selective cultivation and high‐throughput sorting of hydrogen‐producing microorganisms. (A) The core metabolic pathways involved in anaerobic fermentation and hydrogen production. (B) Functional identification of potential anaerobic hydrogen‐producing microorganisms using Venn diagram analysis based on formate dehydrogenase (FDH), acetate kinase (AK), and butyrate kinase (BK). (C) These MAGs were selected using CheckM (completeness > 70% and contamination < 10%). Red pentagrams indicate high‐quality MAGs (completeness > 90% and contamination < 5%). The taxonomic classifications of all MAGs at the phylum level are displayed using different background colors. The taxonomic classifications of all MAGs at the genus level and key functional enzymes for hydrogen production (EC: 1.17.1.9, EC: 2.7.2.1, and EC: 2.7.2.7) for each MAG are shown by heatmaps. Completeness and contamination for each MAG are shown by bar charts. (D) Carbon source utilization spectrum (including various sugars, organic acids, sugar alcohols, and amino acids) of some potential hydrogen‐producing microorganisms. (E) Growth of strains isolated via flow cytometry‐based sorting on solid culture plates.

We screened MAGs containing the functional enzymes (FDH, AK, and BK) for potential anaerobic hydrogen‐producing microorganisms. A Venn diagram analysis on 281 MAGs was performed to identify functional strains with the capability of anaerobic hydrogen production (Figure 2B). A total of 215 MAGs were identified as encoding for potential anaerobic hydrogen producers, each with a completeness greater than 70% and contamination less than 10% (Figure 2C). Among these, 117 high‐quality MAGs (completeness > 95%, contamination < 5%) were identified. The Genome Taxonomy Database (GTDB) toolkit was used to annotate all 215 MAGs. Among them, 209 MAGs were identified as bacteria belonging to 21 phyla, with Bacillota (formerly Firmicutes), Bacteroidota (formerly Bacteroidetes), and Pseudomonadota (formerly Proteobacteria) being dominant. Notably, most reported hydrogen‐producing bacteria belong to these three phyla (Figure 1J) [6, 7]. Six MAGs were assigned to two archaeal phyla (Euryarchaeota and Halobacterota). Although Archaea possess relevant functional enzymes for hydrogen production, they are not typically utilized, as most Archaea use hydrogen primarily as an intermediate metabolite for methane production [10]. Furthermore, hydrogen is produced during desulfurization and denitrification; however, these strains are not primary hydrogen producers and therefore are not classified as hydrogen‐producing microorganisms. Furthermore, this study compared the 215 MAGs with potential anaerobic hydrogen production functions against a database to obtain species information for the top five similarities (Table S2). This provided a basis for the rational design of a selective culture medium.

### Digital cell models guide the formulation of selective medium

Predicting the nutritional requirements and metabolic characteristics of microorganisms based on metagenomic data for targeted sorting of strains remains a significant challenge [11]. Genome‐scale metabolic models (GEMs) are digital cell models that mathematically represent the metabolic capabilities of individual organisms [12]. GEMs integrate genes, reactions, and metabolites related to an organism's metabolic network, which can identify substrate utilization profiles, distinguish nutritional deficiencies in organisms, and predict the optimal growth rates of strains [13]. This provides researchers a quantitative framework to evaluate how microorganisms utilize resources for growth, thereby addressing the limitations of the previous methods.

In this study, GEMs were constructed for 215 MAGs with potential anaerobic hydrogen production functions to identify the substrate utilization spectrum and nutritional deficiencies of each MAG, guiding the preparation of selective culture media. GEMs can be used to predict all metabolic reactions encoded in each MAG, elucidating the utilization, conversion, and exchange patterns of specific substrates under defined environmental conditions [13, 14]. The growth potential of each potential hydrogen producer in media containing 53 different carbon sources was simulated using flux balance analysis (Figures S3 and S4). These carbon sources include various sugars, organic acids, sugar alcohols, and amino acids, chosen based on their presence in biomass saccharification liquid (such as maltose, cellobiose, xylose, and glucose) and general applicability [13]. The simulations indicated that among the 215 MAGs with potential anaerobic hydrogen production functions, 200 MAGs could grow on a single carbon source, whereas the remaining 15 MAGs required two or more carbon sources for normal growth. Figure 2D illustrates the carbon source utilization spectrum of some MAGs. In addition to these carbon sources, we simulated the addition of trace elements to promote the growth of nutrient‐deficient bacteria (Figure S5). Based on the simulation results from the GEMs, a total of 14 selective culture media were formulated (Table S3).

### High‐throughput sorting of hydrogen‐producing microorganisms

Twelve large‐scale biogas plant samples were selectively enriched and cultured using the 14 designed media, followed by high‐throughput sorting with flow cytometry. A total of 81 potential anaerobic hydrogen‐producing strains were successfully sorted, which belonged to 11 phyla, 48 genera, and 66 species (Table S4). Our novel strategy, “prioritizing functional recognition, followed by targeted high‐throughput sorting,” achieved a target strain acquisition rate exceeding 37%. Additionally, the high‐throughput sorting method for anaerobes demonstrated a strain survival rate of over 70% (Figure 2E). Numerous strict anaerobic bacterial strains, including both spore‐forming and non‐spore‐forming types (e.g., *Clostridium* spp. and *Bacteroides* spp.), were successfully isolated. These results further confirm the feasibility and broad applicability of establishing a localized anoxic environment within the flow cytometer's sorting chamber to enable successful sorting of anaerobic bacteria. This approach is straightforward, economical, and practical. However, current high‐throughput sorting technologies predominantly rely on single‐cell separation principles, preventing simultaneous isolation of physically aggregated and functionally interacting microbial consortia. This limitation constitutes a critical bottleneck for microbial sorting—particularly in symbiotic communities—severely constraining microbial resource mining efficiency. Consequently, the development of novel sorting technologies to solve these problems still faces significant challenges.

To overcome this bottleneck, it is essential to gain a deeper understanding of microbial interaction mechanisms. The degree of metabolic niche overlap provides insight into potential competition between organisms by reflecting similarities in resource utilization capabilities [15]. However, the predictive power of niche overlap is limited as it cannot account for positive ecological interactions that can arise from resource partitioning, cross‐feeding, distinct resource acquisition strategies, or evolved cooperation [16, 17]. As a complementary approach, GEMs provide a feasible method to incorporate the effects of these additional mechanisms into predictions of microbial community structure [18]. Integrating GEMs with niche overlap predictions is expected to offer a powerful approach for forecasting interspecies interactions within microbiomes. This will lay a theoretical foundation for the selective cultivation of interacting microbial strains. In addition, the availability of advanced high‐throughput sorting equipment is also critical. Droplet microfluidics is expected to co‐encapsulate target microbial aggregates with essential nutrients in water‐in‐oil (W/O) droplets, enabling the formation of functionalized micro‐units suitable for direct sorting [19, 20]. This strategy opens new avenues for the high‐throughput mining and sorting of previously uncultured or difficult‐to‐culture functional microbial communities.

This study presents a significant advancement in isolating anaerobic hydrogen‐producing microorganisms. Metagenomic data provided extensive species information, facilitating the mining and identification of these strains. Based on MAGs, GEMs were constructed to simulate and predict the carbon source utilization spectrum, nutritional requirements, and metabolic characteristics of these target strains, effectively guiding the preparation of selective culture media. We propose a novel strategy, “prioritizing functional recognition, followed by targeted high‐throughput sorting,” for comprehensive, rapid, and efficient acquisition of the target microorganisms, which offers a valuable and broadly applicable method for mining and sorting of functional microorganisms from diverse natural environments. This provides a novel paradigm for exploiting “uncultivable” microbes and holds significant implications for the industrial application of synthetic microbiomes.

Detailed experimental materials and procedures—including sample collection, 16S rRNA gene high‐throughput sequencing, metagenomic techniques, construction of digital cell models, selective enrichment and sorting of functional strains, and statistical analysis methods—are provided in the Supplementary Information.

## AUTHOR CONTRIBUTIONS

**Jianfeng Liu**: Methodology; validation; conceptualization; funding acquisition; writing—original draft; data curation; resources; visualization; formal analysis. **Wei Xing**: Software; data curation; methodology; formal analysis; visualization. **Xingyang Zhang**: Methodology; visualization; software. **Nengyao Xu**: Methodology; investigation; validation. **Ran Xu**: Methodology; investigation. **Junsha Gong**: Validation; investigation. **Jia Zhang**: Validation; investigation. **Fengai Yang**: Visualization. **Shuang Gao**: Investigation; methodology. **Yanan Hou**: Data curation; methodology. **Yongping Shan**: Data curation; methodology. **Bin Liu**: Methodology; conceptualization. **Qianqian Yuan**: Software. **Aijie Wang**: Supervision; resources; conceptualization; writing—review and editing. **Nanqi Ren**: Conceptualization; writing—review and editing; resources; funding acquisition; project administration. **Cong Huang**: Conceptualization; funding acquisition; writing—review and editing; supervision; project administration. All authors have read the final manuscript and approved it for publication.

## CONFLICT OF INTEREST STATEMENT

The authors declare no conflicts of interest.

## ETHICS STATEMENT

No animals or humans were involved in this study.

## Supporting information


**Figure S1:** Species accumulation box plot at the amplicon sequence variant level.
**Figure S2:** Phylogenetic tree of all metagenome‐assembled genomes (MAGs) with potential anaerobic hydrogen‐producing capacities.
**Figure S3:** Single carbon source utilization spectrum of 200 potential hydrogen‐producing metagenome‐assembled genomes.
**Figure S4:** Multi‐carbon source utilization spectrum of 15 potential hydrogen‐producing metagenome‐assembled genomes.
**Figure S5:** Essential elements of 215 potential hydrogen‐producing metagenome‐assembled genomes.

**Table S1:** Detailed information for completeness and contamination of metagenome‐assembled genomes.
**Table S2:** Top five species of 215 metagenome‐assembled genomes.
**Table S3:** List of carbon sources in all media.
**Table S4:** Taxonomic assignment results of 81 potential hydrogen‐producing bacteria successfully sorted via flow cytometry.
**Table S5:** Information regarding 12 large‐scale biogas project samples in China.

## Data Availability

All the sequencing data have been deposited in GSA under submission number CRA029365 (metagenome sequencing, https://ngdc.cncb.ac.cn/gsa/browse/CRA029365). The data and scripts used are saved in GitHub https://github.com/JianfengLiu1/Biohydrogen_GEMs_iMeta. Supplementary materials (figures, tables, graphical abstract, slides, videos, Chinese translated version, and update materials) may be found in the online DOI or iMeta Science http://www.imeta.science/.
