# Supplementary material for: Metagenomics and digital cell modeling facilitate targeted high‐throughput sorting of anaerobic hydrogen‐producing microorganisms
Source: Imeta. 2025 Sep 21;4(6):e70082. doi: 10.1002/imt2.70082 (PMC12747558; doi:10.1002/imt2.70082)
Supplement: Supplementary file 1 — Figure S1: Species accumulation box plot at the amplicon sequence variant level. Figure S2: Phylogenetic tree of all metagenome‐assembled genomes (MAGs) with potential anaerobic hydrogen‐producing capacities. Figure S3: Single carbon source utilization spectrum of 200 potential hydrogen‐producing metagenome‐assembled genomes. Figure S4: Multi‐carbon source utilization spectrum of 15 potential hydrogen‐producing metagenome‐assembled genomes. Figure S5: Essential elements of 215 potential hydrogen‐producing metagenome‐assembled genomes. [file IMT2-4-e70082-s002.pdf]

**Supplemental Information to**

# Metagenomics and Digital Cell Modeling Facilitate Targeted High-Throughput Sorting of Anaerobic Hydrogen-Producing Microorganisms

**Running title:** High-Throughput Targeted Sorting of Anaerobic Microorganisms

Jianfeng Liu[1], Wei Xing[2,3], Xingyang Zhang[2,4], Nengyao Xu[2], Ran Xu[2], Junsha Gong[2,3], Jia Zhang[2], Fengai Yang[2,3], Shuang Gao[2], Yanan Hou[2,3], Yongping Shan[5], Bin Liu[1,6,7], Qianqian Yuan[2], Aijie Wang[1,8], Nanqi Ren[2,8*], Cong Huang[2*]

[1]Key Laboratory of Environmental Biotechnology, Research Center for Eco-Environmental Sciences, Chinese Academy of Sciences, Beijing 100085, China

[2]National Technology Innovation Center of Synthetic Biology, Tianjin Institute of Industrial Biotechnology, Chinese Academy of Sciences, Tianjin 300308, China

[3]School of Environmental and Municipal Engineering, Tianjin Chengjian University, Tianjin 300384, China

[4]College of Biotechnology, Tianjin University of Science and Technology, Tianjin 300457, China

[5]Key Laboratory of Environmental Nanotechnology and Health Effects, Research Center for Eco-Environmental Sciences, Chinese Academy of Sciences, Beijing 100085, China

[6]State Key Laboratory of Regional Environment and Sustainability, Research Center for

Eco-Environmental Sciences, Chinese Academy of Sciences, Beijing 100085, China

[7]University of Chinese Academy of Sciences, Beijing 100085, China

[8]School of Civil and Environmental Engineering, Harbin Institute of Technology (Shenzhen), Shenzhen 518055, China

*Correspondence: huangc@tib.cas.cn (Cong Huang); rennq@tib.cas.cn (Nanqi Ren);

## Supplementary materials and methods

### Sample collection and DNA extraction

Anaerobic hydrogen production is a crucial biochemical reaction in the biogas fermentation metabolic pathway [1]. In this study, fermentation liquids from 12 large-scale biogas plants were collected for mining and sorting anaerobic hydrogen-producing strains. The experimental design and workflow for mining and sorting anaerobic hydrogen-producing microorganisms is illustrated in Figure 1. Based on the type of waste and location of the biogas plants, the samples were named as follows: YT, ML, NP, NL, JB, JX, CL, CS, ZS, WT, YH, and LF. Detailed information for each sample is provided in Table S5. Total DNA of the microbial community was extracted using the E.Z.N.A.® soil DNA Kit (Omega Bio-tek, Norcross, GA, U.S.) according to the manufacturer's instructions. The quality of the extracted DNA was assessed using 1% agarose gel electrophoresis, and the DNA concentration, purity, and integrity were determined using an Agilent 5400 bioanalyzer. The extracted DNA samples were stored at -20 ºC until further analysis.

### 16S rRNA gene sequencing

The hypervariable region V3-V4 of the bacterial 16S rRNA gene was amplified using PCR with the primer pairs 341F (5′-CCTAYGGGRBGCASCAG-3′) and 806R (5′-CGACTACNNGGGTATCTAAT-3′). The library was constructed using the NEBNext® Ultra™ II FS DNA PCR-free Library Prep Kit (New England Biolabs, Ipswich, MA, USA), and sequencing on the Illumina NovaSeq 6000 platform (Illumina, San Diego, CA, USA). The reads from each sample were assembled using FLASH software (v1.2.11). Quality control of the raw sequencing data was conducted using fastp software (v0.23.1). The DADA2 module in QIIME2

(v2022.2) was employed for noise reduction to obtain the final ASVs and feature tables [2]. Taxonomic assignment of ASVs was performed using the naïve Bayes classifier for taxonomic consensus implemented in QIIME2 with the SILVA 16S rRNA database (v138.1). The Shannon index was used to evaluate the $\alpha$-diversity of a microbial community. The significance among groups was calculated using the Kruskal-Wallis $H$ test (two-sided test, nominal significance level $\alpha = 0.05$) in R (v3.3.1). Significant results were followed by Dunn's post hoc tests with Bonferroni adjustment for multiple comparisons. To determine the variability in the microbial community composition, $\beta$-diversity was evaluated using the Bray-Curtis metric in R (v3.3.1). Non-metric multi-dimensional scaling (NMDS) based on Bray-Curtis distance was used to represent the microbial community structure changes.

**Metagenomic sequencing**

DNA was randomly fragmented to approximately 350 base pairs (bp) using a Covaris M220 ultrasonic disruptor, and sequencing libraries were generated using the NEBNext® Ultra™ DNA Library Prep Kit for Illumina (New England Biolabs, Ipswich, MA, USA). The quantified libraries were pooled and sequenced on Illumina platforms, based on the effective library concentration and data output required. Quality control of the raw data was performed using Readfq (v8) to obtain clean data. A combined assembly of the reads from each sample was prepared using MEGAHIT (v1.0.4) [3]. MetaGeneMark (v2.10) was used for open reading frame prediction within large contigs called Scaftigs (≥ 500 bp) from each sample [4]. Redundancy was eliminated using CD-HIT (v4.6.1) to obtain the non-redundant initial gene catalog [5]. Diamond

(v0.9.9.110) was used to align Unigenes sequences with the MicroNR database for species annotation.

**Binning analysis**

MetaWRAP (v1.2.1) was used to individually assemble the metagenome sequences of each sample and obtain contigs longer than 1500 bp [6]. MetaBAT (v2.12.1) was used to bin the contigs and obtain metagenome-assembled genomes (MAGs) for individual strains [7]. CheckM (v1.0.18) was used to assess the genome integrity and contamination of MAGs, and only those MAGs with integrity ≥ 70% and contamination ≤ 10% were retained [8]. dRep (v3.4.2) was then used for de-redundant clustering to obtain the final de-redundant MAGs. Species annotation of the MAGs was performed using GTDB-TK (v2.3.0) [9]. A phylogenetic tree was constructed using IQtree (v2.0.3) and visualized with iTOL [10]. Default settings were chosen for all tools unless otherwise specified.

**Construction of GEMs**

CarveMe was used to generate genome-scale metabolic models (GEMs) drafts for 215 MAGs [11]. All model drafts were checked for issues, such as exchange reaction boundary errors and net generation problems of energy and metabolites, and corrected using the GEM quality control platform MQC. Carbon source metabolites and exchange reactions were added to each model during the simulation of carbon source utilization capacity to avoid false negatives from the missing exchange reactions. Finally, flux balance analysis was conducted to simulate the growth state and metabolic exchanges of the microorganisms and determine their growth ability and rate on defined culture media [12]. All growth simulations were performed using the COBRA

Toolbox (v0.27.0) in Anaconda (v2.5.4) [13]. Initially, all models were provided with nonlimiting amounts ($v_{max}$ = 1000 mmol g $DW^{-1}$ $h^{-1}$) of minimal medium containing water, ions, and sources of nitrogen, phosphorus, and sulfur, along with the carbon source uptake flux set to 10 mmol g $DW^{-1}$ $h^{-1}$. Subsequently, each carbon source in the set was individually used as the sole carbon source to simulate the growth of each strain, recording the resulting biomass flux and resource uptake flux [14]. Additionally, multi-carbon source simulations were performed for strains unable to grow with a single carbon source, with up to four carbon source combinations tested.

**Shake flask cultivation and selective enrichment**

In this study, 100 mL serum bottles were used for selective enrichment cultures of 12 biogas samples. The culture temperature was maintained at 35 °C, with four pH levels: 5.0, 5.5, 6.0, and 6.5. The minimal medium consisted of NaCl (4 g/L), $K_2HPO_4$ (1.5 g/L), $MgCl_2 \cdot 6H_2O$ (0.1 g/L), $FeSO_4 \cdot 7H_2O$ (0.1 g/L), and L-cysteine (0.5 g/L). The medium was supplemented with the following trace elements: $MnCl_2$ (0.64 mg/L), $ZnSO_4 \cdot 7H_2O$ (4.5 mg/L), $NiCl_2 \cdot 6H_2O$ (0.02 mg/L), $CoCl_2 \cdot 6H_2O$ (3 mg/L), $Na_2MoO_4 \cdot 2H_2O$ (0.4 mg/L), $FeSO_4 \cdot 7H_2O$ (3 mg/L), $H_3BO_3$ (1 mg/L), $CuSO_4 \cdot 5H_2O$ (0.3 mg/L), $Na_2SeO_4 \cdot 5H_2O$ (0.01 mg/L), and $CaCl_2 \cdot 2H_2O$ (3 mg/L). The following vitamins were added: lipoic acid (0.05 mg/L), biotin (0.02 mg/L), niacin (0.35 mg/L), thiamine hydrochloride (0.005 mg/L), *p*-aminobenzoic acid (0.5 mg/L), folic acid (0.02 mg/L), calcium pantothenate (0.05 mg/L), vitamin B12 (0.001 mg/L), and pyridoxine hydrochloride (0.1 mg/L). A list of carbon sources and their concentrations in all media is provided in Table S3. During enrichment, the hydrogen content in the fermentation bottle was monitored to verify production.

**High-throughput sorting and culture**

The agar plates were prepared using the same medium composition as the shake flask cultivation, supplemented with 15 g of Noble agar per liter of medium. The plates were then placed in anaerobic jars to remove oxygen. The mixed bacterial suspension was filtered through a sterile 40 μm cell strainer to remove large particles and cell aggregates. A 5 mL aliquot of the mixed bacterial suspension was diluted to a final cell concentration of approximately 10^6-10^7 cells/mL to ensure the passage of single cells through the detection nozzle. The prepared bacterial suspension was loaded onto a flow cytometer (BD FACSAria Fusion SORP), and data were collected using BD FACSDiva software to generate a two-dimensional scatter plot of forward scatter (FSC) versus side scatter (SSC). Each point on the scatter plot represents a single-cell event, with its position determined by the signal intensities of FSC (X-axis) and SSC (Y-axis). A gating strategy based on defined FSC and SSC signal ranges was applied to accurately select the single-cell population with the desired size characteristics. The agar plate was placed in the sorting chamber of the flow cytometer, where nitrogen was introduced to establish localized anaerobic conditions. Target cells were then accurately sorted as single cells onto the agar surface by deflecting charged droplets, with approximately 100 single colonies deposited per plate. The plates were promptly transferred to anaerobic jars and incubated at 35 °C under anoxic conditions. To evaluate the efficiency of the sorting strategy, we defined the target strain acquisition rate as the proportion of hydrogen-producing strains successfully obtained through high-throughput sorting relative to the number of MAGs annotated with hydrogen-producing potential.

**Functional verification and strain storage**

Once a single colony became visible on the agar plates, it was picked and transferred to an anaerobic test tube for pure culture. Hydrogen content in the anaerobic chamber was measured to verify hydrogen production, and the strains were further identified through polymerase chain reaction (PCR). Finally, the isolated strains were preserved at -80 °C for future use.

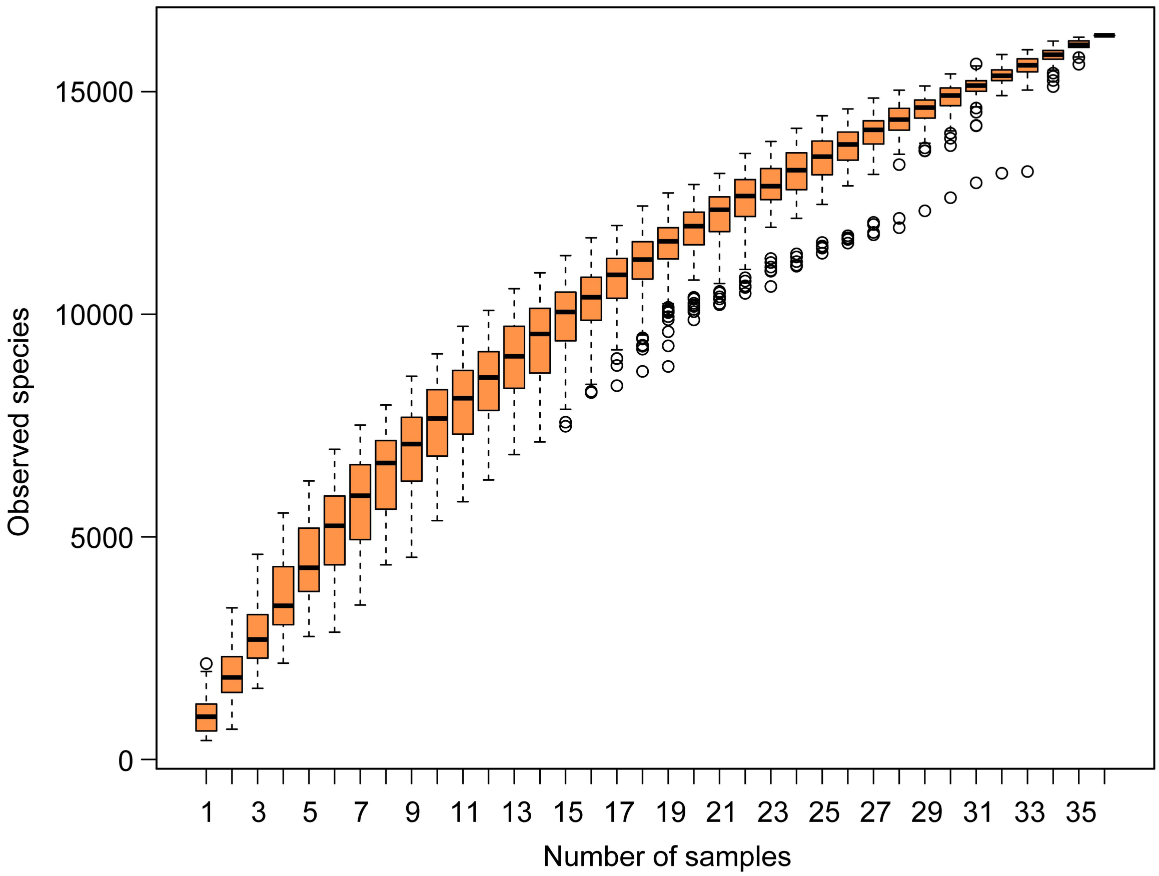


**Figure S1 Species accumulation box plot at the amplicon sequence variant level.** The observed diversity increases with greater sampling effort, and the plateauing of curves indicates sufficient sampling depth.

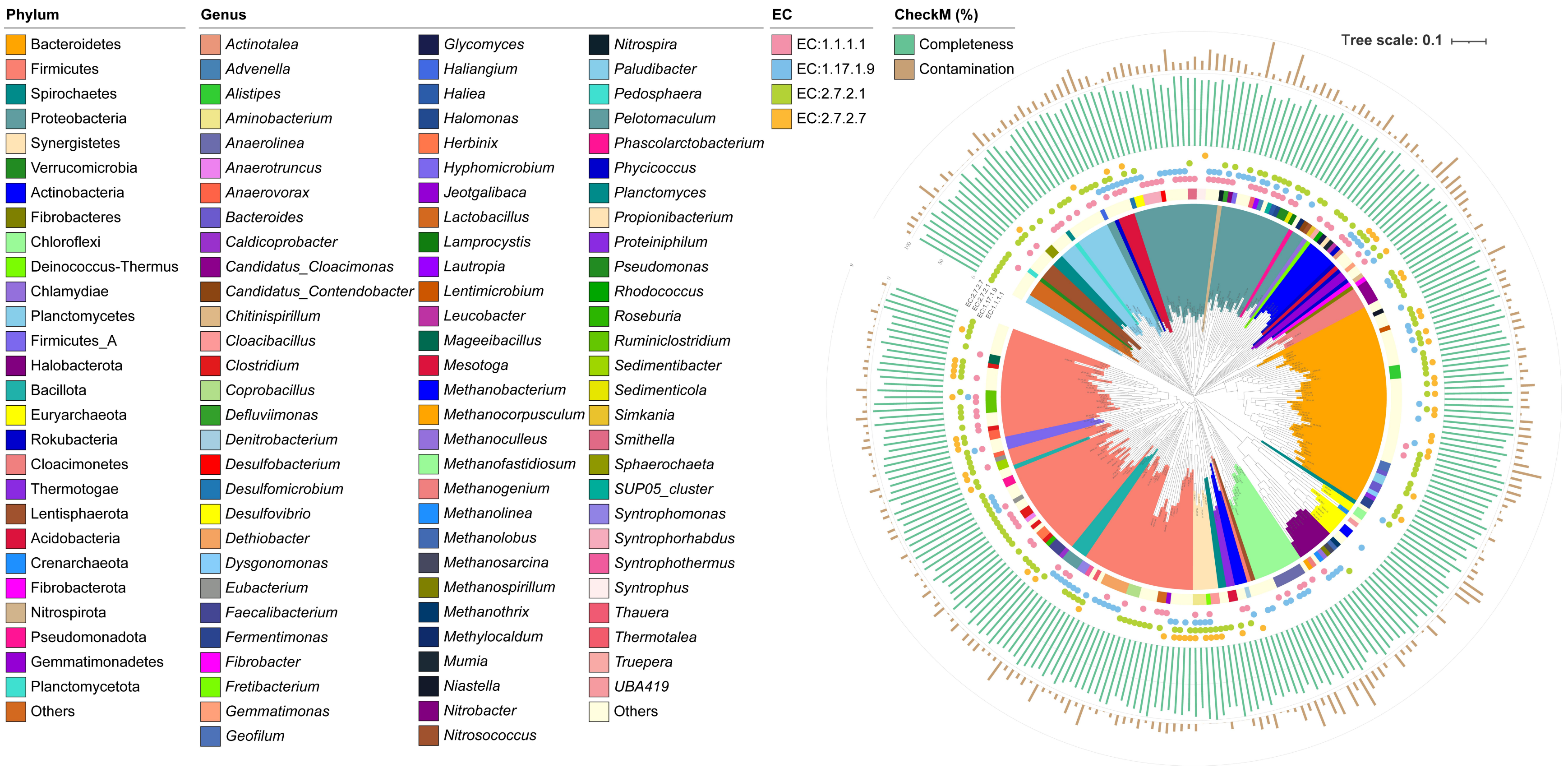


**Figure S2 Phylogenetic tree of all metagenome-assembled genomes (MAGs) with potential anaerobic hydrogen-producing capacities.** These MAGs were selected using CheckM (completeness >70% and contamination <10%). The taxonomic classifications of all MAGs at the phylum level are displayed using different background colors. The taxonomic classifications of all MAGs at the genus level are shown by heatmaps. The key functional enzymes for hydrogen production (EC: 1.17.1.9, EC: 2.7.2.1, and EC: 2.7.2.7) for each MAG are illustrated using dots. Completeness and contamination for each MAG are shown using bar charts.

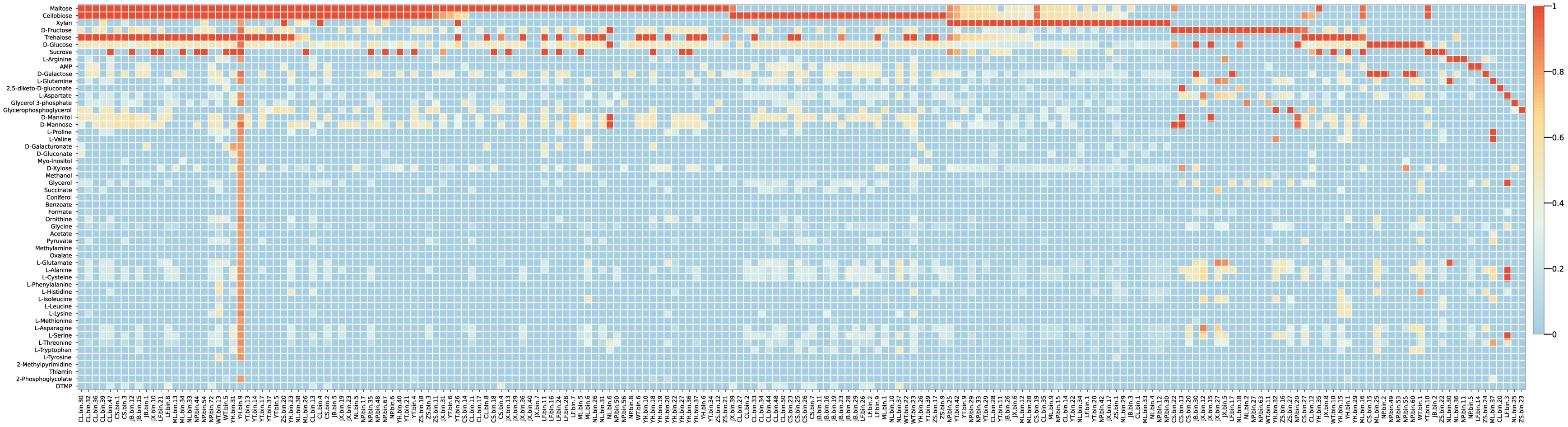


**Figure S3 Single carbon source utilization spectrum of 200 potential hydrogen-producing metagenome-assembled genomes.** The heatmap illustrates the predicted catabolic capabilities of 200 hydrogen-producing MAGs spanning diverse carbon substrates（including sugars, organic acids, sugar alcohols, and amino acids). Rows correspond to individual MAGs, and columns represent specific carbon compounds. Color intensity (a gradient from red to blue) denotes the inferred utilization capacity, with red indicating high potential and blue indicating low or absent metabolic functionality.

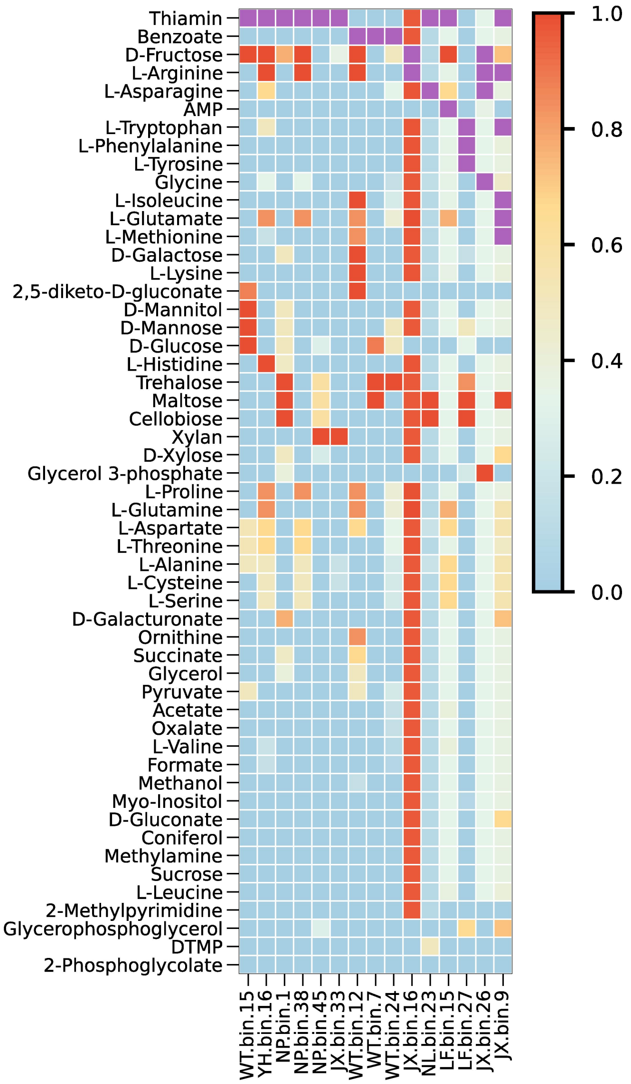


**Figure S4 Multi-carbon source utilization spectrumof 15 potential hydrogen-producing metagenome-assembled genomes.** The heatmap illustrates the predicted catabolic capabilities of 15 hydrogen-producing MAGs spanning diverse carbon substrates （including sugars, organic acids, sugar alcohols, and amino acids). Rows correspond to individual MAGs, and columns represent specific carbon compounds. Color intensity (a gradient from red to blue) denotes the inferred utilization capacity, with red indicating high potential and blue indicating low or absent metabolic functionality. Purple squares represent essential carbon sources.

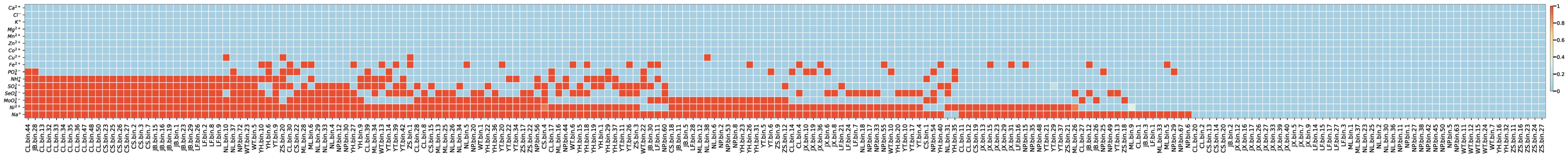

**Figure S5 Essential elements of 215 potential hydrogen-producing metagenome-assembled genomes.** Rows correspond to individual MAGs, and columns represent essential elements. The color gradient (from red to blue) reflects the magnitude of MAGs' demand for each element, where red denotes a high growth demand, while blue indicates a lower dependence on strain growth.

## REFERENCES


1. Sim, Xue Yan, Jian Ping Tan, Ning He, Swee Keong Yeap, Yew Woh Hui, Abdullah Amru Indera Luthfi, Shareena Fairuz Abdul Manaf, Nurul Adela Bukhari, Nur Syakina %J Renewable Jamali, Sustainable Energy Reviews. 2023. "Unraveling the effect of redox potential on dark fermentative hydrogen production." 187: 113755. https://doi.org/10.1016/j.rser.2023.113755

2. Callahan, Benjamin J., Paul J. McMurdie, Michael J. Rosen, Andrew W. Han, Amy Jo A. Johnson, Susan P. Holmes. 2016. "DADA2: High-resolution sample inference from Illumina amplicon data." *Nature Methods* 13: 581-583. https://doi.org/10.1038/nmeth.3869

3. Bolyen, Evan, Jai Ram Rideout, Matthew R. Dillon, Nicholas A. Bokulich, Christian C. Abnet, Gabriel A. Al-Ghalith, Harriet Alexander, et al. 2019. "Reproducible, interactive, scalable and extensible microbiome data science using QIIME 2." *Nature Biotechnology* 37: 852-857. https://doi.org/10.1038/s41587-019-0209-9

4. Karlsson, Fredrik H., Valentina Tremaroli, Intawat Nookaew, Göran Bergström, Carl Johan Behre, Björn Fagerberg, Jens Nielsen, Fredrik Bäckhed. 2013. "Gut metagenome in European women with normal, impaired and diabetic glucose control." *Nature* 498: 99-103. https://doi.org/10.1038/nature12198

5. Li, Weizhong, Adam Godzik, Author Notes. 2006. "Cd-hit: a fast program for clustering and comparing large sets of protein or nucleotide sequences." *Bioinformatics* 22: 1658-1659. https://doi.org/10.1093/bioinformatics/btl158

6. Uritskiy, Gherman V., Jocelyne DiRuggiero, James Taylor. 2018. "MetaWRAP-a flexible pipeline for genome-resolved metagenomic data analysis." *Microbiome* 6: 158. https://doi.org/10.1186/s40168-018-0541-1

7. Kang, Dongwan D., Feng Li, Edward Kirton, Ashleigh Thomas, Rob Egan, Hong An, Zhong Wang. 2019. "MetaBAT 2: an adaptive binning algorithm for robust and efficient genome reconstruction from metagenome assemblies." *Peerj* 7: e7359. https://doi.org/10.7717/peerj.7359

8. Parks, Donovan H., Christian Rinke, Maria Chuvochina, Pierre-Alain Chaumeil, Ben J. Woodcroft, Paul N. Evans, Philip Hugenholtz, Gene W. Tyson. 2017. "Recovery of nearly 8,000 metagenome-assembled genomes substantially expands the tree of life." *Nature Microbiology* 2: 1533-1542. https://doi.org/10.1038/s41564-017-0012-7

9. Parks, Donovan H., Maria Chuvochina, Pierre-Alain Chaumeil, Christian Rinke, Aaron J. Mussig, Philip Hugenholtz. 2020. "A complete domain-to-species taxonomy for Bacteria and Archaea." *Nature Biotechnology* 38: 1079-1086. https://doi.org/10.1038/s41587-020-0501-8

10. Nguyen, Lam T., Heiko A. Schmidt, Arndt von Haeseler, Bui Quang Minh. 2015. "IQ-TREE: a fast and effective stochastic algorithm for estimating maximum-likelihood phylogenies." *Mol Biol Evol* 32: 268-274. https://doi.org/10.1093/molbev/msu300

11. Machado, Daniel, Sergej Andrejev , Melanie Tramontano , Kiran Raosaheb Patil. 2018. "Fast automated reconstruction of genome-scale metabolic models for microbial species and communities." *Nucleic Acids Res* 46: 7542-7553. https://doi.org/10.1093/nar/gky537

12. Orth, Jeffrey D., Ines Thiele, Bernhard Ø. Palsson. 2010. "What is flux balance analysis?" *Nature Biotechnology* 28: 245-248. https://doi.org/10.1038/nbt.1614

13. Ebrahim, Ali, Joshua A. Lerman, Bernhard O. Palsson, Daniel R. Hyduke. 2013. "COBRApy: COnstraints-Based Reconstruction and Analysis for Python." *Bmc Systems Biology* 7: 74. https://doi.org/10.1186/1752-0509-7-74

14. Schäfer, Martin, Alan R. Pacheco, Rahel Künzler, Mirlam Bortfeld-Miller, Christopher M. Field, Evangelia Vayena, Vassily Hatzimanikatis, Julia A. Vorholt. 2023. "Metabolic interaction models recapitulate leaf microbiota ecology." *Science* 381: eadf5121. https://doi.org/10.1126/science.adf5121
